# Impact of atrioventricular node ablation and permanent pacing on clinical outcomes, quality of life, and health care utilization in patients with atrial fibrillation

**DOI:** 10.1016/j.hroo.2026.03.036

**Published:** 2026-04-02

**Authors:** Miriam A. Scheurwater, Gijs J. van Steenbergen, Leonard M. Rademakers, Daniela N. Schulz, Dennis van Veghel, Lukas R.C. Dekker

**Affiliations:** 1 Department of Cardiology, Catharina Heart Center, Catharina Hospital, Eindhoven, The Netherlands; 2 Department of Biomedical Technology, Eindhoven University of Technology, Eindhoven, The Netherlands

**Keywords:** Atrial fibrillation, Atrioventricular node ablation, Ablate-and-pace strategy, Conduction system pacing, Left bundle branch pacing, Health care utilization, Quality of life

## Abstract

**Background:**

Atrioventricular node ablation (AVNA) is a class IIa, level B recommendation in the international atrial fibrillation (AF) guidelines. Although its impact on symptoms is known, effects on health care utilization and quality of life (QoL) remain unclear. Conduction system pacing, particularly left bundle branch pacing (LBBP), may further optimize these outcomes.

**Objective:**

This study aimed to assess the impact of AVNA on clinical outcomes, QoL, and health care utilization.

**Methods:**

Data from patients who underwent AVNA between 2019 and 2024 at a Dutch tertiary referral center were analyzed. Patients received right ventricular pacing, biventricular pacing, or LBBP. Outcomes included (1) clinical outcomes, (2) 36-item Short Form Health Survey QoL scores at baseline and 1-year follow-up, and (3) health care utilization per patient-year 1 year before up to 1 year after AVNA.

**Results:**

Of 248 patients (median age 77 years; 44.4% male), most received LBBP (62.5%), followed by biventricular (19.8%) and right ventricular pacing (17.7%). Procedural complications were minimal (0.8%). 90-day mortality was 4.0% (n = 10), 1-year mortality was 10.5% (n = 26), and deaths were related to heart failure (46.2%) or noncardiac causes (53.8%). QoL improved in 67.0% and deteriorated in 14.4%. After AVNA, health care utilization decreased significantly across all domains: outpatient clinic visits (−27.5%), emergency department visits (−72.1%), Holter analyses (−84.2%), electrocardiograms (−52.5%), electrical cardioversions (−98.2%), additional AF ablations (−83.3%), and pacemaker/implantable cardioverter-defibrillator upgrades (−81.8%; all *P* < .001).

**Conclusion:**

AVNA is a safe intervention associated with a significant reduction in health care utilization and improved QoL. These findings support AVNA as a valuable strategy for managing AF in an increasingly resource-constrained health care environment.

## Graphical abstract

**Figure:**
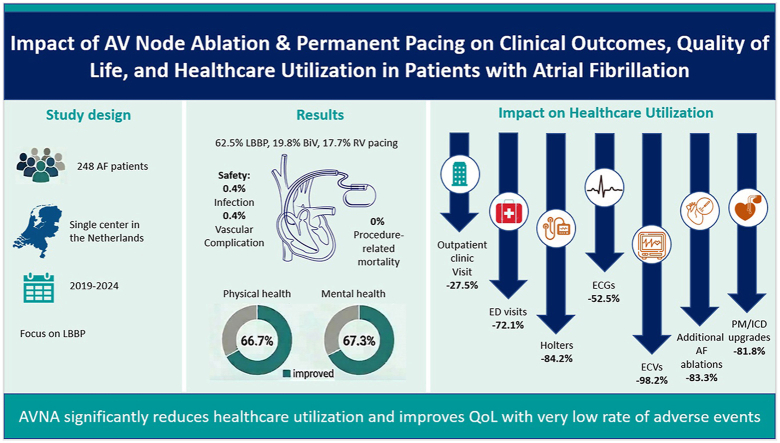

**Key Findings**

▪ Atrioventricular node ablation (AVNA) was associated with a substantial reduction in health care utilization in patients with refractory atrial fibrillation (AF), including fewer outpatient clinic visits, emergency department visits, Holter analyses, electrocardiograms, electrical cardioversions, additional AF ablations, and pacemaker/implantable cardioverter-defibrillator upgrades.
▪ Quality of life improved in approximately two-thirds of patients 1 year after AVNA in both physical and mental health domains.
▪ AVNA demonstrated a favorable safety profile, with very low procedural complication rates and no mortality directly related to the procedure.
▪ Left bundle branch pacing provided similar benefits in health care utilization and quality of life as conventional pacing strategies, despite its greater technical complexity.
▪ Favorable outcomes were also observed in younger patients (younger than 65 years), suggesting that AVNA may be considered in carefully selected younger individuals with therapy-refractory AF.

## Introduction

Atrioventricular node ablation (AVNA) and pacemaker (PM) implantation are classified as a class IIa recommendation with level of evidence B in the 2023 American College of Cardiology/American Heart Association/Heart Rhythm Society and 2024 European Society of Cardiology (ESC) guidelines for the management of atrial fibrillation (AF) (“ablate-and-pace” strategy).^1^^,^^2^ This recommendation is based on previous research in a cohort of patients with refractory AF and a high symptom burden, where AVNA demonstrated low complication rates and low long-term mortality.^3^, ^4^, ^5^, ^6^, ^7^, ^8^, ^9^ However, the most common complication is pacing-induced cardiomyopathy, occurring in more than 10% of patients with chronic right ventricular (RV) pacing.^10^ Conduction system pacing (CSP), more specifically left bundle branch pacing (LBBP), has emerged as a potential alternative, offering more physiological ventricular activation and possibly mitigating the risk of heart failure.^11^, ^12^, ^13^, ^14^, ^15^ Research among patients with AF has shown persistently high rates of health care utilization, which decreases only slightly after pulmonary vein isolation (PVI) but remains elevated primarily owing to repeated electrical cardioversions (ECVs), hospitalizations, and repeated left atrial ablation procedures, including substrate modification beyond PVI such as linear lesions or posterior wall isolation.^16^^,^^17^ However, the effects of AVNA on health care utilization and quality of life (QoL) remain unclear, especially in the light of LBBP.

For maintaining accessible high-quality health care in an era of rising health care costs and a global shortage of hospital personnel, reducing health care utilization needs to become a critical priority.^18^ Frequent hospital visits, recurrent interventions, and prolonged hospitalizations place an immense burden on both health care systems and patients.^19^, ^20^, ^21^, ^22^, ^23^ Moreover, optimizing QoL for patients with AF requires minimizing their dependence on the health care system by implementing effective, durable treatment strategies.^24^ The “ablate-and-pace” strategy may provide a means to achieve these goals for patients with therapy-refractory AF, but a comprehensive evaluation of its impact on health care utilization and patient outcomes is necessary given that the procedure induces irreversible damage to the cardiac conduction system. Although LBBP may provide electrical and hemodynamic benefits over traditional RV pacing, its implantation is technically more complex and could introduce additional health care utilization.^12^

This study aimed to assess the impact of AVNA in patients with uncontrolled AF on clinical outcomes, QoL, and health care utilization, with a specific focus on the effects of LBBP as a pacing strategy after AVNA. By addressing these factors, this study sought to contribute to the optimization of care for patients with refractory AF while alleviating strain on health care resources.

## Methods

### Study setting and patient population

The current study used a retrospective cohort design, focusing on patients who underwent AVNA between January 2019 and November 2024 at a large tertiary referral center in the Netherlands. This institution serves as the intervention center within a hub-and-spokes model^25^ and is a high-volume center for AF catheter ablation procedures.^26^ Furthermore, the center was an early adopter of LBBP, initiating the first procedures in 2020 as an alternative to both conventional RV and biventricular pacing. Eligible participants were adults aged 18 years and older who had undergone AVNA to treat AF. For patients who underwent more than 1 AVNA during the study period, only the first procedure was included in the analysis; subsequent redo procedures were excluded. The research reported in this paper adhered to the ethical principles of the Declaration of Helsinki. According to the institutional guidelines of the study center, formal institutional review board approval and individual informed consent were not required for this retrospective analysis of pseudonymized data.

#### PM implantation procedures

All procedures were performed under local anesthesia with administration of 2-g intravenous cefazolin for perioperative prophylaxis. Direct oral anticoagulants were withheld 24 hours before the procedure, whereas vitamin K antagonists were continued if the international normalized ratio remained <3.0. The standard venous approach was the cephalic vein using a modified Seldinger technique. If cephalic access was unsuccessful, axillary or subclavian venous puncture was used as an alternative. The implanting physician selected the pacing modality.

##### Conventional RV pacing procedure

Pacing leads were placed in the septal or apical region of the RV, as decided by the implanting physician.

##### Biventricular lead implantation

After cannulation of the coronary sinus and venography, selection of the quadripolar left ventricle lead was based on the decision of the implanting physician. The lead was preferably placed in a basal position in a lateral or posterolateral vein, whereas the RV lead was positioned in the RV septum or apex.

##### LBBP procedure

LBBP was performed using the SelectSecure Model 3830 74-cm pacing lead (Medtronic, Minneapolis, MN) and the C315HIS delivery sheath (Medtronic). The implantation technique was published previously.^12^ LBBP was confirmed when the paced QRS morphology demonstrated a (pseudo) right bundle branch block morphology, a left ventricular activation time that shortened abruptly with increasing output and not exceeding 95 ms, and a V6–V1 interpeak interval of >33 ms.^27^

#### AVNA procedure

All procedures were performed under local anesthesia. Direct oral anticoagulants were continued, whereas vitamin K antagonists were continued if the international normalized ratio remained <3.0. Vascular access was obtained via a single, echocardiography-guided puncture of the femoral vein using a standard sheath. An ablation catheter was positioned at the His-bundle region under fluoroscopic guidance. Mapping of the His bundle was performed, after which radiofrequency energy was applied using a nonirrigated, non-thermocool ablation catheter with a 4-mm tip at the proximal His region until complete atrioventricular (AV) block was achieved. If residual conduction was observed, additional energy applications were delivered until stable AV block was confirmed. Procedural success was defined as complete and permanent AV block after a waiting period of at least 15 minutes. A pressure bandage was applied for groin management.

### Data collection and study outcomes

Data on baseline characteristics, clinical outcomes, QoL, and health care utilization were retrieved from electronic patient files by specialized data analysts and defined according to relevant guidelines from the American College of Cardiology/American Heart Association/Heart Rhythm Society, ESC, and the Netherlands Heart Registration.^1^^,^^2^^,^^28^ The latter coordinates the Dutch national cardiac registry and ensures data quality through standardized collection protocols, rigorous validation, and regular audits.^29^ Baseline characteristics included age, gender, body mass index, left ventricular ejection fraction (LVEF), renal function, left atrial volume index, preoperative mitral valve regurgitation, CHA_2_Ds_2_-VASc score, previous ablation, type of AF (paroxysmal, persistent, or longstanding persistent), and type of pacing.

The following clinical outcomes were included as primary outcomes: mortality at 90 days and 1 year, bleeding complications during admission, thromboembolic complications at 72 hours, pneumothorax during admission, infection within 90 days, vascular complications at 30 days, cardiac tamponade within 30 days, and event-free survival.^30^ In cases of mortality, the cause of death was obtained from medical records.

QoL is routinely assessed at baseline (before the ablation) and at 1-year follow-up using the 36-item Short Form Health Survey (SF-36 version 2).^31^

Health care utilization was assessed based on care registration data used for reimbursement from insurance companies. Owing to the mandatory health insurance system in the Netherlands, the completeness of health care utilization is ensured. For the present study, a previously described method for the selection of relevant care activities by van Steenbergen et al^32^ was adopted. In short, through a step-wise approach, a multidisciplinary panel of medical experts, nonclinical professionals, and patient representatives selects care activities that are relevant for evaluation of AF care based on the following criteria: relevance to patients, prevalence in clinical settings, potential for modification by health care providers, and significant financial implications. These parameters can be addressed as patient-relevant cost drivers and are divided into diagnostics, hospital visits, and treatments. As a result, the following patient-relevant cost drivers were selected: Holter analyses and electrocardiograms (diagnostics); outpatient clinic visits, hospital admissions, and emergency department visits (hospital visits); and ECVs, additional AF ablation, and PM or implantable cardioverter-defibrillator (ICD) upgrades (treatments). PM/ICD upgrades were only included in the pre-AVNA analysis if they occurred more than 3 months before AVNA to avoid potential confounding from the standard preprocedural management phase.

### Statistical analysis

Baseline characteristics, clinical outcomes, QoL, and health care utilization were expressed as absolute and relative frequencies for categorical data and as mean values with corresponding standard deviation or median with interquartile range (IQR) for continuous data depending on the normality of data distribution (normal vs non-normal distribution, respectively). Missing values in baseline characteristics were considered missing at random and did not exceed 5%. A χ^2^ with Monte Carlo simulation or Kruskal-Wallis test with Bonferroni correction was used to make a comparison among the types of pacing. A Kaplan-Meier survival curve was used to display the trend in all-cause 1-year mortality after AVNA.

For each participant, standardized domain scores (0–100 scale) were calculated across the SF-36 domains. These domains were then grouped into 2 composite scores reflecting physical health and mental health. A change score was calculated for each composite by subtracting baseline from follow-up. QoL was categorized as improved if the difference exceeded +5 points, deteriorated if the score declined by ≥5 points, and equal if the change was within ±5 points. Only patients who completed at least 50% of the SF-36 questionnaire at baseline and at 1-year follow-up were included in the QoL analysis.

To evaluate the impact of AVNA on health care utilization, 2 distinct time periods were distinguished for each patient: the period 1 year before the ablation (pre-AVNA period) and the period 1 year thereafter (post-AVNA period). These periods were analyzed individually and compared with one another using a paired *t* test. To account for variations in follow-up time owing to mortality within 1 year after AVNA, health care utilization was expressed as mean per patient-year using a group-level correction factor. This factor was calculated by dividing the total patient-years (365), if all patients had survived, by the actual observed patient-years (342), resulting in a factor of .94. All health care utilization was divided by this factor to normalize the data to a full patient-year. This group-level approach was preferred over individual correction to minimize the disproportionate impact of extreme outliers. We conducted a subgroup analysis of patients who deteriorated in QoL, according to the pacing strategy, and patients younger than 65 years to evaluate whether the outcomes in this specific group were consistent with findings in the overall population.

*P* < .05 was considered significant for all analyses. Analyses were performed using SPSS version 29 (IBM, Chicago, IL).

## Results

### Participants

Between 2019 and November 2024, a total of 248 AVNAs were performed (see Table 1). The median age was 77 years (IQR 71–81 years), and 44.4% were male. Most patients (57.1%) had a normal LVEF, 29.6% a mildly reduced, and 13.4% a severely reduced LVEF. A total of 119 patients (48.0%) underwent previous AF ablation. The type of AF was divided into paroxysmal (25.0%), persistent (49.2%), or longstanding persistent (25.8%). Most patients received LBBP (62.5%), followed by biventricular (19.8%) and conventional RV pacing (17.7%). The use of LBBP increased over time, as shown in Supplemental Figure 1. Baseline characteristics differed significantly among the pacing groups (Supplemental Table 1). Patients with biventricular pacing were more often male than those with RV or LBBP (*P* = .003). LVEF was markedly lower in the biventricular pacing group (median 31%; IQR 25–40) than both RV pacing (median 55%; IQR 50–55) and LBBP groups (median 55%; IQR 45–55) (*P* < .001). Similarly, the biventricular pacing group had significantly lower estimated glomerular filtration rate than the LBBP group (*P* < .001). The CHA_2_Ds_2_-VASc score was slightly lower in the LBBP group than the biventricular pacing group according to the IQR (*P* = .022). Previous AF ablation was more frequent in the LBBP group (56.1%) than in the RV (40.9%) and biventricular pacing groups (28.6%) (*P* = .002). Paroxysmal AF was more common in the RV pacing group, persistent AF in the LBBP group, and longstanding persistent AF in the biventricular pacing group (*P* = .007).

**Table 1:** Baseline characteristics of the total cohort (N = 248)

Baseline characteristics	Total (N = 248)
Men, n (%)	110 (44.4)
Age, y, median (IQR)	77 (71–81)
BMI, kg/m^2^, median (IQR)	26.8 (23.7–29.6)
LVEF, median (IQR)	55 (40–55)
LVEF, n (%)	
LVEF >50	141 (57.1)
LVEF 30–50	73 (29.6)
LVEF <30	33 (13.4)
eGFR, ml/min per 1.73 m^2^, median (IQR)	56.1 (41.0–73.8)
Renal insufficiency, n (%)	
eGFR 30–59	120 (48.4)
eGFR 15–29	21 (8.5)
eGFR <15	3 (1.2)
Left atrial volume index, mL/m^2^, median (IQR)	45 (38–57)
Preoperative mitral valve regurgitation, n (%)	
None/mild	184 (75.4)
Moderate	51 (20.9)
Severe	9 (3.7)
CHA_2_Ds_2_-VASc, median (IQR)	4 (3–5)
Previous AF ablation, n (%)	119 (48.0)
Type AF, n (%)	
Paroxysmal	62 (25.0)
Persistent	122 (49.2)
Longstanding persistent	64 (25.8)
Type of pacing, n (%)	
LBBP	155 (62.5)
Conventional RV pacing	44 (17.7)
Biventricular pacing	49 (19.8)

AF = atrial fibrillation; BMI = body mass index; eGFR = estimated glomerular filtration rate; IQR = interquartile range; LBBP = left bundle branch pacing; LVEF = left ventricular ejection fraction; RV = right ventricular.

### Clinical outcomes

**Figure 1:**
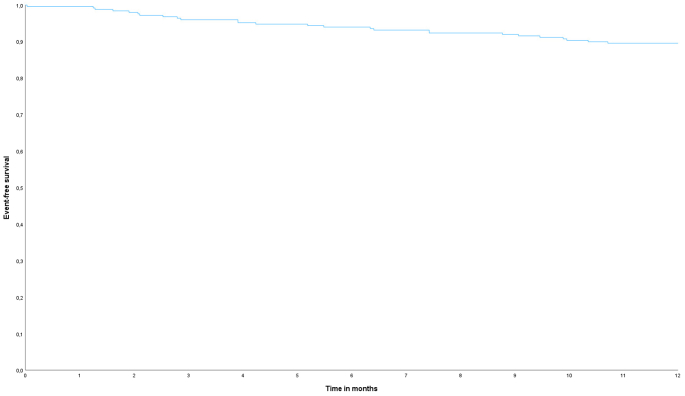
Kaplan-Meier curve of event-free survival.

1 patient (0.4%) developed an infection within 90 days of AVNA, and a vascular complication occurred in only 1 patient (0.4%) 30 days after AVNA (see Table 2). Mortality at 90 days occurred in 10 patients (4.0%), and 1-year mortality occurred in 26 patients (10.5%). Event-free survival is presented in Figure 1. Mortality was not related to AVNA in any of the patients. 12 (46.2%) were attributed to preexisting heart failure, and 14 (53.8%) to noncardiac causes, including sepsis, pulmonary embolism, or malignancy.

**Table 2:** Clinical outcomes and QoL

Clinical outcomes and QoL	Total (N = 248)
Mortality, 90 d, n (%)	10 (4.0)
Mortality, 1 y, n (%)	26 (10.5)
Bleeding complications during admission, n (%)	0 (0.0)
Thromboembolic complication, <72 h, n (%)	0 (0.0)
Pneumothorax during admission, n (%)	0 (0.0)
Infection, <90 d, n (%)	1 (0.4)
Vascular complications, <30 d, n (%)	1 (0.4)
Tamponade, <30 d, n (%)	0 (0.0)
QoL, n (%)	
Physical health	
Improved QoL	70 (66.7)
Equal QoL	16 (15.2)
Deteriorated QoL	19 (18.1)
Mental health	
Improved QoL	70 (67.3)
Equal QoL	23 (22.1)
Deteriorated QoL	11 (10.6)

QoL = quality of life.

### QoL

Of the 248 patients included in the study, 105 patients (42.3%) completed the QoL questionnaire at both baseline and 1-year follow-up and were included in the QoL analysis. Most patients experienced an improved QoL compared with baseline, in both physical health (66.7%) and mental health (67.3%). A small group of patients experienced deteriorated QoL in physical health (18.1%) and mental health (10.6%).

### Health care utilization

**Figure 2:**
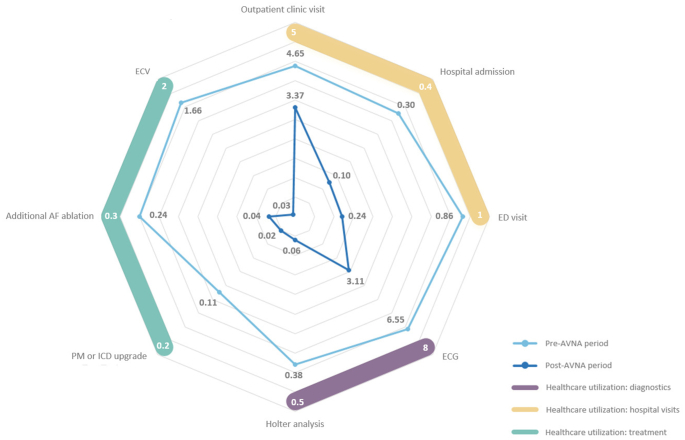
Spider chart depicting average health care utilization per patient-year divided in diagnostics (*purple*), hospital visits (*yellow*), and treatment (*green*) for the pre-AVNA period (*light blue line*) and the post-AVNA period (*dark blue line*). The *outer ring* indicates a reference value, and the *center* is 0 for every parameter. AF = atrial fibrillation; AVNA = atrioventricular node ablation; ECG = electrocardiogram; ECV = electrical cardioversion; ICD = implantable cardioverter-defibrillator; PM = pacemaker.

In the year after AVNA, health care utilization significantly declined across all domains (see Table 3). A spider chart of mean health care utilization per patient-year before and after AVNA across diagnostics, hospital visits, and treatments is presented in Figure 2. Patients required fewer Holter analyses, with a mean reduction of .32 per patient-year (−84.2%; *P* < .001). Similarly, the number of electrocardiograms decreased by a mean of 3.44 per patient-year (−52.5%; *P* < .001), and ECVs by 1.63 per patient-year (−98.2%; *P* < .001). In addition, the need for repeat AF ablations (−83.3%; *P* < .001) and device-related procedures, such as PM/ICD upgrades, also declined (−81.8%; *P* < .001). 2 patients required a repeat AVNA and underwent ECVs during the waiting period. Outpatient clinic visits decreased by a mean of 1.28 per patient-year (−27.5%; *P* < .001), emergency department visits by .62 per patient-year (−72.1%; *P* < .001), and hospital admissions by .20 (−66.7%; *P* < .001). When analyzing health care utilization in relation to 1-year mortality, a significant difference was observed in the frequency of additional AF ablations (*P* = .003). Although most of the cohort showed a substantial reduction in additional AF ablations after AVNA (mean .27 to .02), patients who died within 1 year experienced an increase in the rate of additional AF ablations (mean .04 to .16). In addition, those with 1-year mortality exhibited a more pronounced decline in the frequency of electrocardiograms (*P* = .030) than those who remained alive. No significant differences in change of health care consumption were found for outpatient clinic visits (*P* = .135), hospital admissions (*P* = .739), emergency department visits (*P* = .961), Holter analyses (*P* = .169), PM/ICD upgrades (*P* = .265), or ECVs (*P* = .463).

**Table 3:** Health care utilization of the total cohort 1 year before AVNA and 1 year after AVNA (depicted as mean health care utilization per patient-year)

Health care utilization	Before AVNA (n = 248)	After AVNA (n = 248)	*P* value
	Mean per patient-year	Mean per patient-year	
Holter analysis	.38	.06	<.001∗
Electrocardiogram	6.55	3.11	<.001∗
Electrical cardioversion	1.66	.03	<.001∗
Additional AF ablation	.24	.04	<.001∗
PM/ICD upgrade	.11	.02	<.001∗
Outpatient clinic visit	4.65	3.37	<.001∗
Hospital admission	.30	.10	<.001∗
Emergency department visit	.86	.24	<.001∗

AF = atrial fibrillation; AVNA = atrioventricular node ablation; ICD = implantable cardioverter-defibrillator; PM = pacemaker.

∗ Statistically significant.

### Subgroup analysis of patients with deteriorated QoL

Most patients who deteriorated in physical health were patients who received conventional RV pacing, and most of those who deteriorated in mental health were patients who received LBBP (see Supplemental Table 2). These patients did not differ notably from the overall cohort in terms of baseline characteristics, and none experienced complications or mortality. Patients with deteriorated QoL showed a significantly greater reduction in the number of ECVs after AVNA than those with an equal or improved QoL (*P* = .025). No significant differences were observed between the deteriorated and nondeteriorated QoL groups regarding the change in other health care utilization.

### Subgroup analysis of patients who received LBBP

More than half of the study population received LBBP. Mortality occurred in 5 patients at 90 days and in 13 patients at 1 year. All deaths were attributed to preexisting heart failure (n = 5; 38.5%) or noncardiac causes (n = 8; 61.5%). QoL improvements were comparable with those in the total cohort. Health care utilization decreased significantly for all domains (*P* < .001) (see Supplemental Table 3).

### Subgroup analysis of patients younger than 65 years

No deaths or complications occurred among the 27 patients younger than 65 years, corresponding to an event-free survival of 100% (see Supplemental Table 4). Most patients reported improved QoL in physical health (68.4%) and mental health (73.7%), consistent with the overall cohort. A small proportion reported deteriorated QoL in physical health (15.8%; n = 3), and only 1 patient reported deteriorated mental health (5.3%). Health care utilization significantly decreased for Holter analysis (*P* = .045), ECVs (*P* < .001), and additional AF ablations (*P* = .001) (see Supplemental Table 5).

## Discussion

The current study demonstrated that AVNA in patients with refractory AF is a safe and effective intervention, with minimal procedural complications, improved QoL for most patients, and a reduction in health care utilization across all domains, which was not the result of substitution of care. Furthermore, AVNA combined with LBBP was associated with a similar reduction in health care utilization compared with conventional RV or biventricular pacing. Although the pacing groups differed in baseline characteristics, these findings suggest that the adoption of LBBP, despite its perceived technical complexity and learning curve, can offer an effective alternative to maintaining the benefits of resource efficiency without increasing the long-term health care burden. Combined, these findings indicate that AVNA can add value to the disease trajectory of the patient with AF, particularly in the context of rising health care demand and limited staffing capacity. However, given the retrospective single-center design of the present study, these findings should be interpreted with caution. Although our results suggest that AVNA may represent a valuable treatment strategy for selected patients with refractory AF, future prospective and comparative studies are needed to determine the optimal timing of AVNA relative to medical therapy or repeated left atrial ablations. On a systemic level, these findings suggest that the impact of AVNA extends to a collective scale. By reducing the burden of repeated interventions and hospitalizations, this strategy helps maintain accessible high-quality health care and alleviates pressure on hospital personnel. To support such interventions, payment models should transition toward value-based reimbursement, prioritizing long-term resource efficiency and patient independence over the volume of repeated procedures.^33^

The current ESC guidelines highlight that the available evidence for AVNA primarily pertains to older patients.^2^ In the present study, we also evaluated outcomes in a small subgroup of younger patients (younger than 65 years). Although the number of patients in this subgroup was limited (n = 27), no deaths or complications were observed and improvements in QoL and reductions in health care utilization were consistent with the overall cohort. To the best of our knowledge, data describing outcomes of AVNA specifically in younger patients remain scarce, and therefore, our findings provide preliminary insight into this underreported population. Although these observations should be interpreted cautiously given the small sample size and the absence of a control group, they suggest that AVNA may also represent a feasible treatment strategy for selected younger patients with refractory AF. Larger prospective studies are needed to better define the role and timing of AVNA in younger patient populations.

The reduction in health care utilization after AVNA was previously shown to be more significant than the reduction observed after left atrial ablation procedures. Data from the German Ablation Registry revealed significantly higher cardiovascular rehospitalization rates in PVI patients than AVNA patients (31% vs 18%; *P* < .001).^34^ This difference is likely driven by the high recurrence rates of AF even after left atrial ablation, with ranges between 31% and 54% described, often necessitating repeated ECVs, hospitalizations, or redo procedures.^17^^,^^35^^,^^36^ In our cohort, the pre-AVNA period also reflected substantial rhythm-control management, with 48% of patients having undergone a previous AF ablation and frequent rhythm-control interventions in the year preceding AVNA, including ECVs (1.66 per patient-year) and additional AF ablations (0.24 per patient-year). In addition, once an ablate-and-pace strategy is adopted, rhythm-control interventions are generally no longer pursued, which may inherently contribute to the observed reduction in rhythm-related health care utilization. In AVNA, the ventricular rate is entirely regulated by the PM, which may result in patients experiencing no or minimal symptoms of AF. Disease-specific QoL questionnaires also showed more improvement in QoL after AVNA than after left atrial ablation. Nevertheless, our study also identified a subset of patients with deterioration of QoL, which warrants further study. Although these findings suggest that AVNA contributes to reduced health care utilization and improved QoL, it should be considered whether the observed reduction in health care utilization is partly influenced by provider behavior, such as altered follow-up intensity or referral thresholds. However, our observation of a significant decrease in unplanned care—including emergency department visits and unscheduled hospitalizations—suggests that the reduction reflects a genuine clinical improvement and stabilization of the patient’s condition, rather than solely a shift in planned follow-up strategies. Future research should focus on the optimal timing of AVNA within the AF disease trajectory, particularly after 1 or more unsuccessful left atrial ablations. Prospective studies are needed to determine whether earlier transition to an ablate-and-pace strategy, rather than pursuing repeated left atrial procedures, could further optimize health care utilization and long-term QoL.

The observed vascular complication rate and infection rate of 0.4% and the absence of mortality directly related to the procedure underscore AVNAs procedural safety, even in an elderly population with a median age of 77 years. A low complication rate is also described elsewhere and is considered lower or similar to left atrial ablation and medical therapy.^5^^,^^37^^,^^38^ The 1-year mortality rate of 10.5% aligns with previous studies and highlights the extensive underlying comorbidities in this cohort.^34^^,^^38^ Long-term follow-up is deemed favorable after AVNA because it has been associated with sustained improvements in symptom control and QoL and a significant reduction in long-term health care utilization, including lower rates of rehospitalization for cardiovascular events.^38^^,^^39^

One limitation of the ablate-and-pace strategy is the potential development of PM-induced cardiomyopathy owing to left ventricular dyssynchrony, affecting up to 50% of patients with RV pacing.^40^ Although RV pacing effectively controls heart rate, reduces hospitalizations, and improves QoL, it may contribute to adverse ventricular remodeling in some patients.^5^ The relatively high proportion of LBBP in our study can be explained by our center being an early adopter of the CSP technique. Since its introduction in 2020, the use of LBBP has gradually increased, despite the absence of formal guidelines or consensus recommendations at that time. Emerging evidence suggests that CSP strategies such as His-bundle pacing and LBBP may better preserve physiological ventricular activation and could reduce the risk of pacing-induced cardiomyopathy compared with conventional RV pacing.^14^^,^^41^ However, the present study was not designed to evaluate long-term ventricular function, and no longitudinal LVEF data were available. Therefore, the potential protective effects of LBBP on ventricular function cannot be assessed in this cohort. Our findings instead demonstrate that the use of LBBP did not result in increased health care utilization compared with conventional pacing strategies, despite its perceived procedural complexity. Based on the latest evidence, CSP may be preferred over RV or biventricular pacing depending on LVEF: in patients with preserved LVEF (>40%), CSP may prevent deterioration of ventricular function, whereas, in those with reduced LVEF (≤40%), CSP seems superior to biventricular pacing in improving heart failure outcomes.^41^ Future studies with longer follow-up and serial echocardiographic assessment are needed to further clarify the long-term clinical benefits of CSP after AVNA.

### Limitations

The retrospective design of the current study is inherently associated with a lesser level of evidence, and the use of data from a single center might limit the generalizability of results. Upcoming trials might provide a higher level of evidence for the position of AVNA than PVI.^42^ QoL analyses were restricted to the 42.3% of patients who completed the SF-36 questionnaire at both baseline and 1-year follow-up. Given that patients who died during follow-up could not complete the 1-year questionnaire and additional data were missing for other reasons, the QoL findings may be subject to survivor and responder bias. EQ-5D data are not routinely collected at our center; thus, a formal cost-effectiveness analysis could not be performed. The current study might also be limited by the relatively short-term follow-up of patients given that pacing-induced cardiomyopathies require longer to develop.^10^

## Conclusion

AVNA was associated with a significant reduction in health care utilization, an increase in QoL, and a very low rate of adverse events. AVNA should be considered as additional treatment in patients with AF, particularly combined with LBBP, including in younger patients, in light of recent advancements in CSP.

## Disclosures

The authors have no conflicts of interest to disclose.
