# Supplementary material for: Impact of atrioventricular node ablation and permanent pacing on clinical outcomes, quality of life, and health care utilization in patients with atrial fibrillation
Source: Heart Rhythm O2. 2026 Apr 2;7(7):1344–52. doi: 10.1016/j.hroo.2026.03.036 (PMC13390001; doi:10.1016/j.hroo.2026.03.036)
Supplement: Supplementary Material [file mmc1.docx]

**SUPPLEMENTARY MATERIALS**

**Supplementary materials figure 1.** Annual distribution of pacing modalities in AVNA patients.

AVNA: atrioventricular node ablation; LBBP: left bundle branch pacing; RV: right ventricular.

**Supplementary materials table 1.** Baseline characteristics divided into type of pacing.

| **Baseline characteristics** | **LBBP (n = 155)** | **Conventional RV-pacing (n = 44)** | **Biventricular pacing (n = 49)** | **p-value** |
| --- | --- | --- | --- | --- |
| Male, *N (%)* | 69 (44.5%) | 11 (25.0%) | 30 (61.2%) | **.003** |
| Age, *median (IQR)* | 77 (72-82) | 80 (75-84) | 73 (64-81) | .051 |
| BMI, kg/m^2^, *median (IQR)* | 26.9 (24.1-30.0) | 25.8 (22.8-30.1) | 26.2 (23.5-29.3) | .357 |
| LVEF, *median (IQR)* | 55 (45-55) | 55 (50-55) | 31 (25-40) | **<.001** |
| LVEF, *N (%)* |  |  |  | **<.001** |
| LVEF >50 | 99 (64.3%) | 33 (75.0%) | 9 (18.4%) |  |
| LVEF 30 - 50 | 47 (30.5%) | 10 (22.7%) | 16 (32.6%) |  |
| LVEF <30 | 8 (5.2%) | 1 (2.3%) | 24 (49.0%) |  |
| eGFR, ml/min, *median (IQR)* | 59.0 (49.3-76.4) | 48.9 (35.3-57.5) | 41.7 (32.6-65.8) | **<.001** |
| Renal insufficiency, *N (%)* |  |  |  | .125 |
| eGFR 30 - 59 | 69 (44.5%) | 25 (56.8%) | 26 (53.1%) |  |
| eGFR 15 - 29 | 8 (5.2%) | 3 (6.8%) | 10 (20.4%) |  |
| eGFR <15 | 1 (0.6%) | 1 (2.3%) | 1 (2.0%) |  |
| Left atrial volume index, mL/m^2^, *median (IQR)* | 45 (37-57) | 45 (38-55) | 52 (38-58) | .389 |
| Preoperative mitral valve regurgitation, *N (%)* |  |  |  | .084 |
| None/mild | 115 (75.2%) | 34 (79.1%) | 35 (72.9%) |  |
| Moderate | 36 (23.5%) | 6 (13.9%) | 9 (18.8%) |  |
| Severe | 2 (1.3%) | 3 (7.0%) | 4 (8.3%) |  |
| CHA_2_Ds_2_-VASc, *median (IQR)* | 4 (3-5) | 4 (4-6) | 4 (4-6) | .**022** |
| Previous AF ablation, *N (%)* | 87 (56.1%) | 18 (40.9%) | 14 (28.6%) | **.002** |
| Type AF, *N (%)* |  |  |  | **.007** |
| Paroxysmal | 32 (20.6%) | 19 (43.2%) | 11 (22.5%) |  |
| Persistent | 86 (55.5%) | 16 (36.4%) | 20 (40.8%) |  |
| Longstanding persistent | 37 (23.9%) | 9 (20.4%) | 18 (36.7%) |  |

AF: atrial fibrillation; BMI: body mass index; eGFR: estimated glomerular filtration rate; IQR: interquartile range; LBBP: left bundle branch pacing; LVEF: left ventricular ejection fraction; RV; right ventricular.

**Supplementary materials table 2.** Outcomes of quality of life depicted as percentage of patients per pacing strategy.

|  | **LBBP, *N (%)*** | **Conventional RV-pacing, *N (%)*** | **Biventricular pacing, *N (%)*** |
| --- | --- | --- | --- |
| Physical health  Improved QoL  Equal QoL  Deteriorated QoL | 51 (66.2%)  12 (15.6%)  14 (18.2%) | 7 (58.3%)  2 (16.7%)  3 (25.0%) | 12 (75.0%)  2 (12.5%)  2 (12.5%) |
| Mental health  Improved QoL  Equal QoL  Deteriorated QoL | 49 (64.5%)  18 (23.7%)  9 (11.8%) | 9 (75.0%)  2 (16.7%)  1 (8.3%) | 12 (75.0%)  3 (18.8%)  1 (6.2%) |

LBBP: left bundle branch pacing; RV-pacing: right ventricular pacing; QoL: quality of life.

**Supplementary materials table 3.** Healthcare utilization one year pre-AVNA and one year post-AVNA for patients who received left bundle branch pacing. Depicted as mean healthcare utilization per patient-year.

| **Healthcare utilization** | **Pre-AVNA (n = 155)** | **Post-AVNA (n = 155)** | **p-value** |
| --- | --- | --- | --- |
|  | **Mean per patient-year** | **Mean per patient-year** |  |
| Holter analysis | .43 | .04 | **<.001** |
| Electrocardiogram | 6.82 | 3.63 | **<.001** |
| Electrical cardioversion | 1.55 | .02 | **<.001** |
| Additional AF ablation | .31 | .04 | **<.001** |
| PM/ICD upgrade | .09 | .01 | **<.001** |
| Outpatient clinic visit | 4.20 | 2.54 | **<.001** |
| Hospital admission | .31 | .05 | **<.001** |
| Emergency department visit | .81 | .16 | **<.001** |

AF: atrial fibrillation; AVNA: atrioventricular node ablation; ICD: implantable cardioverter defibrillator; PM: pacemaker.

**Supplementary table 4.** Clinical outcomes and quality of life for patients aged <65.

| **Clinical outcomes and quality of life** | **Total (n = 27)** |
| --- | --- |
| Mortality 90 days, *N (%)* | 0 (0.0%) |
| Mortality one-year, *N (%)* | 0 (0.0%) |
| Bleeding complications during admission, *N (%)* | 0 (0.0%) |
| Thromboembolic complication <72 hours, *N (%)* | 0 (0.0%) |
| Pneumothorax during admission, *N (%)* | 0 (0.0%) |
| Infection <90 days, *N (%)* | 0 (0.0%) |
| Vascular complications <30 days, *N (%)* | 0 (0.0%) |
| Tamponade <30 days, *N (%)* | 0 (0.0%) |
| Quality of life, *N (%)*  Physical health  Improved QoL  Equal QoL  Deteriorated QoL  Mental health  Improved QoL  Equal QoL  Deteriorated QoL | 13 (68.4%)  3 (15.8%)  3 (15.8%)  14 (73.7%)  4 (21.1%)  1 (5.3%) |

QoL: quality of life.

**Supplementary materials table 5**. Healthcare utilization one year pre-AVNA and one year post-AVNA for patients aged <65. Depicted as mean healthcare utilization per patient-year.

| **Healthcare utilization** | **Pre-AVNA (n = 27)** | **Post-AVNA (n = 27)** | **p-value** |
| --- | --- | --- | --- |
|  | **Mean per patient-year** | **Mean per patient-year** |  |
| Holter analysis | .67 | .16 | **.045** |
| Electrocardiogram | 5.44 | 3.79 | .056 |
| Electrical cardioversion | 1.85 | .12 | **<.001** |
| Additional AF ablation | .51 | .04 | **.001** |
| PM/ICD upgrade | .12 | .04 | .327 |
| Outpatient clinic visit | 4.77 | 3.67 | .149 |
| Hospital admission | .20 | .08 | .265 |
| Emergency department visit | .51 | .16 | .153 |

AF: atrial fibrillation; AVNA: atrioventricular node ablation; ICD: implantable cardioverter defibrillator; PM: pacemaker.
