# Supplementary figures and images for: Impact of atrioventricular node ablation and permanent pacing on clinical outcomes, quality of life, and health care utilization in patients with atrial fibrillation
Source: Heart Rhythm O2. 2026 Apr 2;7(7):1344–52. doi: 10.1016/j.hroo.2026.03.036 (PMC13390001; doi:10.1016/j.hroo.2026.03.036)

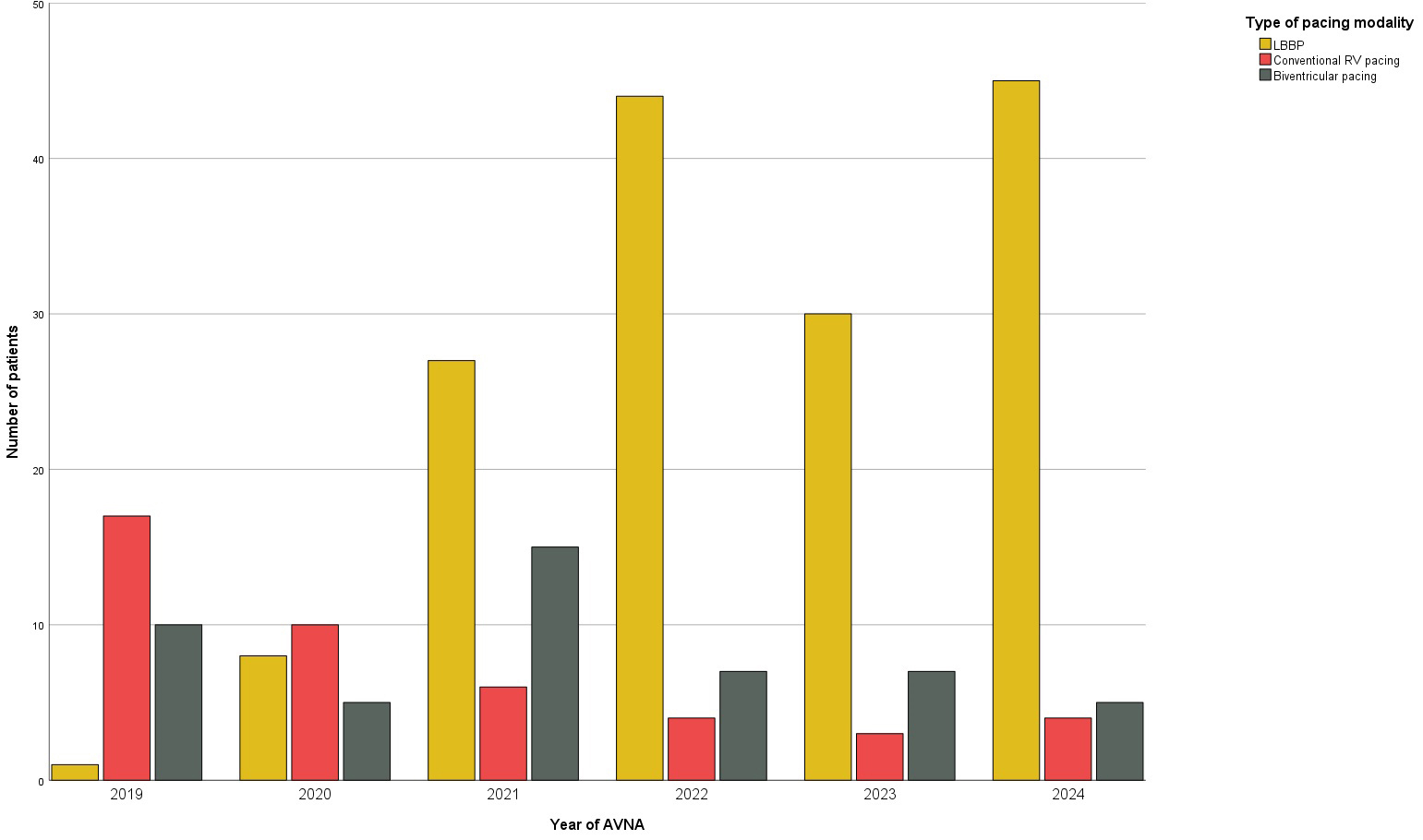

Supplement: Supplementary file 2 [file figs1.jpg]
